# Silver Nanoparticles as Potential Antiviral Agents

**DOI:** 10.3390/pharmaceutics13122034

**Published:** 2021-11-29

**Authors:** Zubair Ahmed Ratan, Fazla Rabbi Mashrur, Anisha Parsub Chhoan, Sadi Md. Shahriar, Mohammad Faisal Haidere, Nusrat Jahan Runa, Sunggyu Kim, Dae-Hyuk Kweon, Hassan Hosseinzadeh, Jae Youl Cho

**Affiliations:** 1 Department of Biomedical Engineering, Khulna University of Engineering & Technology, Khulna 9203, Bangladesh; zubairahmed@bme.kuet.ac.bd (Z.A.R.); rabbi.mashrur@gmail.com (F.R.M.); anishachhoan17@gmail.com (A.P.C.); 2 School of Health and Society, University of Wollongong, Wollongong, NSW 2500, Australia; hassanh@uow.edu.au; 3 Department of Materials Science and Engineering, University of California-Davis, Davis, California, CA 95616, USA; sadimdshahriar@mse.kuet.ac.bd; 4 Department of Materials Science and Engineering, Khulna University of Engineering and Technology, Khulna 9203, Bangladesh; 5 Department of Soil, Water and Environment, University of Dhaka, Dhaka 1000, Bangladesh; mfaisal.dhaka@gmail.com; 6 Sylhet MAG Osmani Medical College, Sylhet 3100, Bangladesh; nusratruna17@gmail.com; 7 Department of Integrative Biotechnology, Sungkyunkwan University, Suwon 16419, Korea; sukim590@skku.edu (S.K.); dhkweon@skku.edu (D.-H.K.); 8 Department of Biocosmetics, Sungkyunkwan University, Suwon 16419, Korea; 9 Biomedical Institute for Convergence at SKKU (BICS), Suwon 16419, Korea

**Keywords:** silver nanoparticles, viral infection, antiviral activity, virus

## Abstract

Since the early 1990s, nanotechnology has led to new horizons in nanomedicine, which encompasses all spheres of science including chemistry, material science, biology, and biotechnology. Emerging viral infections are creating severe hazards to public health worldwide, recently, COVID-19 has caused mass human casualties with significant economic impacts. Interestingly, silver nanoparticles (AgNPs) exhibited the potential to destroy viruses, bacteria, and fungi using various methods. However, developing safe and effective antiviral drugs is challenging, as viruses use host cells for replication. Designing drugs that do not harm host cells while targeting viruses is complicated. In recent years, the impact of AgNPs on viruses has been evaluated. Here, we discuss the potential role of silver nanoparticles as antiviral agents. In this review, we focus on the properties of AgNPs such as their characterization methods, antiviral activity, mechanisms, applications, and toxicity.

## 1. Introduction

Viral diseases are one of the greatest threats to humanity, causing a number of pandemics throughout history. Viruses are thought to have originated from multiple ancient cells and to have co-existed with the ancestors of modern cells [1]. They come in a variety of shapes and sizes in nature, ranging from 20 to 900 nanometers, and are able to act as vectors in animals, plants, bacteria, and fungus [2]. Viruses can be transmitted vertically (from mother to child) or horizontally (from person to person) [3]. They can spread through direct contact, exchange of saliva, coughing, or sneezing; while some viral transmission pathways require sexual contact, others are transmitted through the fecal-oral route via contaminated food or water [3]. Moreover, some viruses can cause persistent infections that can lead to cancer or acquired immunodeficiency such as hepatitis viruses or human immunodeficiency virus (HIV). The need for antiviral drugs is related to the clinical importance and prevalence of viral infections [4]. Viruses are pathogenic agents that cause significantly increased morbidity and death worldwide. For instance, approximately 2 million people around the world face death due to viruses annually [5]. Their highly contagious nature and lack of efficient control methods have serious health consequences [6]. However, with the help of vaccination programs, some viral diseases have been eradicated, such as smallpox in 1979 [7]. In recent years, some viral vaccines have been developed; new vaccines and drugs are required to further decrease the burden [8,9].

**Figure 1:**
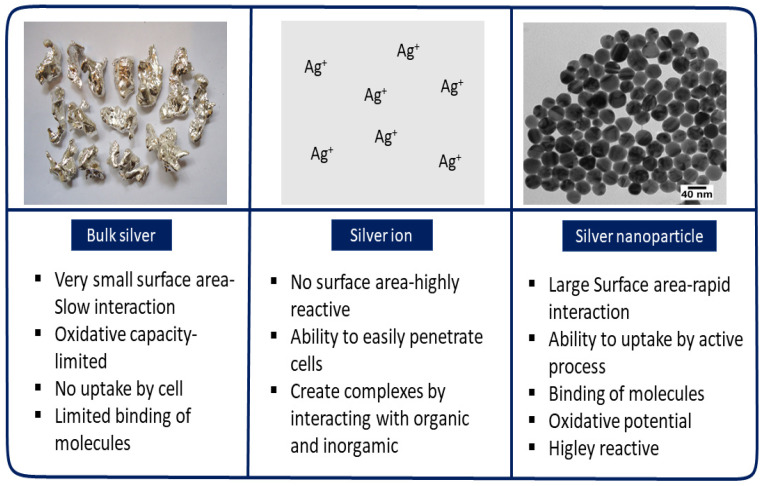
The fundamental variations between the characteristics of silver in ionic, nanoparticulate, and bulk forms.

In recent years, nanotechnology has demonstrated remarkable advances in the battle against viruses. In biomedicine, nanotechnology has led to revolutionary biomolecular systems that can detect certain types of cells, viruses, bacteria, and fungi by developing individual components with nanoscale characteristics (100 nm). Nanoscale particles have been introduced as antiviral agents for their physical (e.g., plasmonic resonance, fluorescent enhancement) and chemical (e.g., catalytic activity enhancement) properties, which are derived from a large quantity of surface atoms and high area/volume ratio. When the diameter of a particle decreases, the available surface area of the particle increases significantly; as a result, compared to bulk materials or ions, distinctive nanoparticle qualities increase (Figure 1). Unique features such as these lead to better and safer medications, tissue-conscious therapies, tailored nanomedicines, and early diagnosis and disease prevention [10]. Many nano-based compounds have been developed to enhance targeted delivery and treatment effectiveness of antiviral medicines [11]. Since the COVID-19 pandemic began, the interest in nanotechnology among researchers has expanded considerably [12,13,14,15].

Metallic nanoparticles (NPs) offer a wide range of applications. Silver [16,17], titanium [18], copper [19], zinc oxide, and iron oxide [20] are examples of metallic NPs that have been used in consumer items such as disinfectants [15], personal protective equipment [21], and cosmetics [22]. Among the metallic NPs, silver nanoparticles (AgNPs) have attracted considerable attention because of their unique properties [23]. AgNPs have several advantages compared to conventional chemical drugs that target viruses [24,25,26]; specifically, they can attach to viral surface glycoproteins and enter host cells, where they exhibit virucidal activity by interacting with the viral genome [16].

This study presents a review of the antiviral activity of AgNPs against viruses along with their possible antiviral mechanism. This work also includes corresponding properties, characterization techniques, toxicity, and applications of AgNPs.

## 2. Synthesis of Silver Nanoparticles

**Figure 2:**
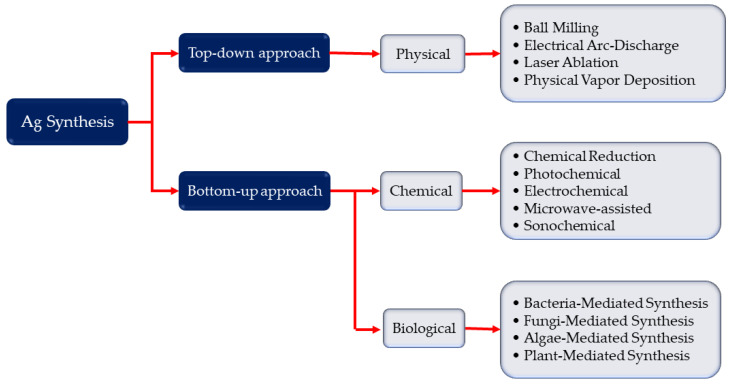
Synthesis of silver nanoparticles: top-down and bottom-up approaches, i.e., physical, chemical, and biological synthesis processes independently. The top-down technique refers to the creation of complex clusters and obtained nanoparticles from molecular components, whereas the bottom-up approach relates to the synthesis of metal nanoparticles from bulk materials.

AgNP synthesis is primarily separated into two processes: top-down and bottom-up approaches (Figure 2). The top-down approach refers to the synthesis of metal NPs from bulk materials using a variety of physical forces, including ball milling (use mechanical energy), grinding, and crushing; electrical arc-discharge and laser ablation method use electrical energy; and vapor condensation use thermal energy [27]. These methods may produce NPs between 10 and 100 nm in size, allowing for the production of pure nanoparticles without the need of chemical additions. Physically produced NPs may have a homogeneous particle size distribution and great purity. Despite the absence of chemical reagents that may harm humans and the environment, the physical technique poses a significant problem in preventing agglomeration due to the lack of stabilizers or capping agents. Furthermore, these procedures necessitate the use of sophisticated equipment as well as external energy. The bottom-up technique, on the other hand, uses nucleation and growth processes to build complex clusters from molecular components in order to create NPs [27,28]. Chemical and biological synthesis are two popular bottom-up techniques that may both produce NPs by lowering the precursor salt, and are also frequently used in the literature Ag NPs synthesis against virus [29,30]. Alternative energies, such as photochemical [29,31], electrochemical [32], microwave-assisted [33], and sonochemical processes [30,34], can be used with chemical synthesis. This method produces NPs between 1–400 nm which act as an antiviral agent. Despite the fact that the chemical approach is used to swiftly create varied forms of NPs, the inclusion of toxic chemical additions may restrict the medicinal uses of NPs. To compensate for the inadequacies of the chemical technique, the biological method has been proposed as an alternative in some works against virus [35,36]. Exopolysaccharide, cellulose, and enzymes are common macromolecular components in bacteria, fungus, and algae [37], as are organic components in plant extracts such as enzymes, alcohol, flavonoids, alkaloids, quinines, terpenoids, and phenolic compounds [37,38,39,40]. The size of Ag NPs against virus is between 16 to 120 nm. Biological synthesis is a cost-effective, ecologically friendly, easy, and dependable method. However, the components on nanoparticle surfaces must be taken into account.

## 3. Properties of Silver Nanoparticles

Nanomaterials show unique properties in comparison to the corresponding bulk materials, the nanoparticle is not only determined by chemical composition but also their morphological and surface properties. Based on their properties, nanomaterials are used in different purpose such as drug delivery, bio-imaging or disease diagnosis [41].The properties of silver nanoparticles are given below:

### 3.1. Shape and Crystallinity

Different fabrication methods allow the generation of AgNPs having various sizes and shapes, such as prisms, spheres, wires, plates, or rods. For example, a photo-induced method was utilized to transform Ag nano-spheres into triangular nano-prisms [42,43,44]. Studies conducted by Mirkin and Murphy’s groups have yielded a seeding technique for Ag nano-prism synthesis and control of edge length [45,46,47]. Another study reported an altered polyol process where ethylene glycol plays not only the role of solvent, but also the role of reducing agent to produce varied AgNP shapes, including pentagonal nanowires, right bipyramids, and nano-cubes with tunable corner truncation [48]. Among techniques to synthesize Ag triangular nano-plates, microwave heating has been used widely [49,50,51,52].

### 3.2. Melting Temperature

The melting temperature property of metallic nanoparticles is substantially lower than that of the bulk materials [53]. The melting temperature of bulk silver is constant at 960 °C. The melting point of AgNPs is lower than that of bulk silver and varies according to NP diameter. This phenomenon can be explained by the Gibbs−Thomson effect [54,55]. As the particle size approaches the nano-size range, the surface area to volume ratio increases. Because of the surface energy contribution resulting from this increased ratio, the melting point of the nanoparticles has an approximately linear relationship with the inverse of particle radii [56]. The Gibbs−Thomson effect is the higher tendency of smaller particles to sinter or Ostwald ripen such that the total free energy is reduced [57], causing surface sintering or melting of nanoparticles to occur at a lower temperature.

### 3.3. Optical Properties

No recognized organic or inorganic chromophores interact with visible light as efficiently as do AgNPs. These nanoparticles have effective light-interaction cross-sections up to 10 times larger than their geometric cross-section, which means that they have much greater light capture capability than the intensity physically incident on them [43,58]. In metallic silver, when the conduction of a huge number of electrons is restricted because of the smaller dimensions than their mean free path as well as their distinctive frequency dependent of the dielectric function (both real and imaginary parts), a light interaction takes place. These two characteristics together give rise to the phenomenon called surface plasmon resonance (SPR). Not only the structure and proportions of the NPs, but also the dielectric function of the adjacent environment control and regulate the frequency and strength of the resonance [42]. Surface plasmons arise at the boundary between a conductor and an insulator through collective excitation of the electrons. Coherent oscillation of conduction band electrons occurs when they are acted upon by an external electromagnetic field. The electron cloud from the nuclei is dislocated by this phenomenon, which later results in a charge distribution on the surfaces of the particles. The collective oscillation of the electron cloud dislocated to the particle surface is called the SPR [59].

### 3.4. Electrical Properties

Characterization of the high-frequency electrical behavior of AgNP-based conductors up to 220 GHz showed that, at frequencies over 80 GHz, electrical losses from samples fabricated from AgNPs are lower than those of similar conductors fabricated using thick-film silver conductors fired at much higher temperatures. The lower loss observed at higher frequencies is attributed to the lower surface roughness conferred by NPs due to better packing; AgNPs have the potential to be utilized in low-temperature fabrication of antennas and better-performing sub-THz metamaterials [60].

## 4. Characterization of AgNPs

Characterization is an important step for determining the morphology, size, shape, and surface chemistry of the nanoparticles. The physicochemical properties have a significant impact on their biological application. In order to ensure the safety issue is it is very important to characterize the prepared nanoparticles before application [61,62]. Characterization techniques used for AgNPs are UV-visible (UV-Vis) spectroscopy, dynamic light scattering (DLS), scanning electron microscopy (SEM), transmission electron microscopy (TEM), atomic force microscopy (AFM), Fourier transform infrared (FTIR) spectroscopy, X-ray diffraction (XRD), energy dispersive spectroscopy (EDS) or energy-dispersive X-ray spectroscopy (EDXS), X-ray photoelectron spectroscopy (XPS), diffuse reflectance spectroscopy (DRS), zeta potential analysis, and correlative light and electron microscopy (CLEM) [63,64].

UV-Vis spectroscopy detects the formation of AgNPs from the color shift resulting from SPR. Because of SPR, the color of the aqueous solution turns yellowish-brown [65]. An absorption peak at around 430 nm usually indicates AgNPs [66] when using light in the wavelength range of 300–800 nm [67].

For nanomaterials and micro-scale materials, SEM has been used widely to obtain information about topography and chemical structure. In this process, a high-energy electron beam is used over a large area of the specimen to provide a high-resolution image [68].

TEM is useful for morphological analysis of nanoparticles when they are arranged in a thin film [69]. Along with delivering size-related information of the nanoparticles, TEM also provides an insight into the chemical structure of the specific nanoparticles by producing lattice images [70]. An obvious benefit of TEM over SEM is its 1000-fold higher resolution [71].

AFM is another technique for analyzing the surface morphology of nanoparticle samples. This technique implements a highly sensitive cantilever with a sharp tip that traverses the surface while the deflection is recorded and generates an image with atomic-level data [68,72].

DLS is a special technique [73] with which the average diameter and size of nanoparticles and their surface charges can be computed [74]. Usually, a scattering laser light is passed through a colloidal solution or within nanoparticles. Later, this movement is measured by DLS and analyzed according to Brownian motion [68].

In the synthesis of AgNPs, various reducing and capping agents might play significant roles depending on the method used. FTIR sheds light on the agents involved by identifying the functional groups attached to the surfaces of the nanoparticles [75]. To identify phases and determine the crystal structure of the sample, XRD is used. The identification is performed based on diffraction patterns after beams of X-rays from the source of the XRD machine are directed onto the mounted sample [76]. EDS or EDXS is used to perform elemental analysis of the AgNPs based on a strong signal corresponding to silver. However, signals due to other elements such as carbon and oxygen also can be detected because of the various biomolecules attached to the nanoparticles [77]. The XPS technique allows the usage of X-rays on a nanomaterial under some specific conditions like an ultra-high vacuum environment and elucidates the details about the inner structural arrangement of the nanoparticles. The measurement of the generated kinetic energy and the movement of the electrons due to the incidence of the X-rays produces the desired XPS spectrum. Again, this process has the advantage of identifying sample contaminants [68]. Zeta potential analysis is another method for determining the stability of nanomaterials in suspensions. This process measures the active surface electric charge of the colloidal particles under the presence of an electric field and has a proportional relationship with particle stability [68]. The DRS method is applied to evaluate the optical properties of a nanocrystal. This process can determine the bandgaps of nanoparticles and the absorption shift for doped materials along with the usual optical reflectance, absorption, and transmittance properties [78,79]. In the CLEM method, samples placed on a microscopic glass slide are coated distinctly to prepare them for easy evaluation. After acquiring the coordinates of the specified markers, they are stored in software to calibrate and determine correlations among them [80,81].

## 5. Antiviral Mechanisms of AgNPs

**Figure 3:**
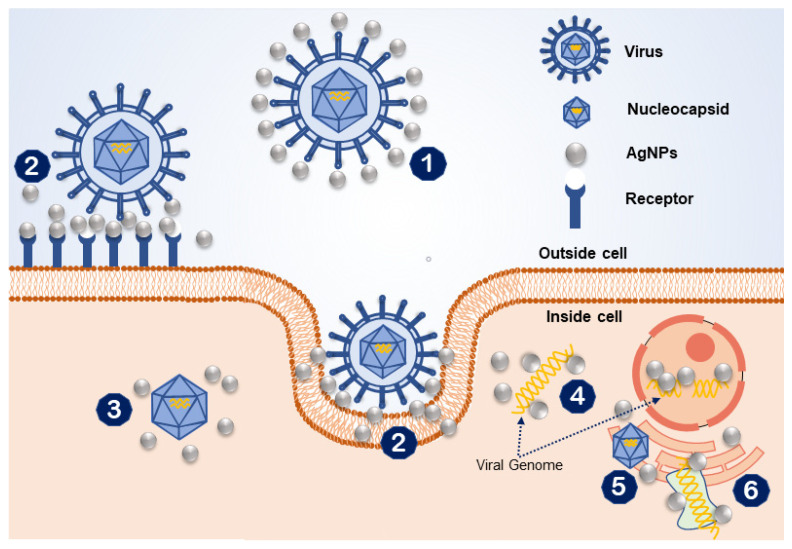
An illustration of the possible antiviral mechanisms of silver nanoparticles (AgNPs): (1) interaction of AgNPs with the viral surface; (2) interface with the cell membrane while blocking the viral attachment; (3) inhibition of cellular pathways of the virus; (4) interaction with viral genome; (5) interaction to inhibit viral genome replication; (6) inhibition of cellular factors (i.e., protein synthesis) necessary for viral replication.

The precise mechanism through which AgNPs kill viruses remains uncertain. AgNPs can be applied to prevent multiple viral infections either by inhibiting infection of the virus in cells or by directly inactivating the viruses, such as with herpes simplex virus (HSV), respiratory syncytial virus, and adenovirus type 3 [30,82,83]. AgNPs have high antiviral activity because of their large surface area, which facilitates contact with viral particles (Figure 1). Usually, antiviral agents act directly on viral particles by binding to viral coat proteins and impeding structural interactions or functions. Here, the size of AgNPs plays a critical role in viral-nanoparticle interactions [84]. A possible antiviral mechanism of AgNPs is illustrated in Figure 3. Initially, AgNPs interact with the viral surface, which ultimately leads to destruction of the viral genomic material or prevents it from penetrating the cell membrane. AgNPs also attach to the viral entity to interfere with the interaction of the virus with the cell membrane. AgNPs also act as inhibitors of the nucleocapsids of the viral entity inside the cell. Moreover, AgNPs interact with the viral genomic material and inhibiting genome replication inside the host cell. Lastly, they interrupt cellular factors like protein synthesis to inhibit replication of the viral entity. Although different types of metals have antiviral properties, AgNPs are the most effective; they have shown high antiviral efficacy against different types of viruses regardless of virus family.

## 6. Activity of AgNPs against Viruses

In recent years, many researchers have proven the efficacy of AgNPs to inhibit viral entities. In this section, we discuss the activity of AgNPs against different viral entities.

### 6.1. Adenovirus

Adenoviruses are non-enveloped, double-stranded DNA (dsDNA) viruses [85]. Infections caused by these viruses can result in respiratory, ocular, or gastrointestinal diseases and can be endemic in the pediatric population. These viruses cause infections worldwide throughout the year, and currently available drugs against adenoviruses are limited [86]. For this reason, there is high demand for novel anti-adenovirus drugs. Silver nanoparticles were shown to have an inhibitory effect on adenovirus type 3 (Ad3) in an in vitro experiment. Spherical AgNPs were fabricated via a chemical redox method by adding 1% tannic acid to AgNO_3_ solution. This reduced the viral fluorescence intensity in Ad3-infected HeLa cells. Disruption of the Ad3 structure and viral DNA damage are possible causes of this inhibitory effect [87].

### 6.2. Hepatitis B

Hepatitis B virus (HBV), a member of the family *Hepadnaviridae* and genus *Orthohepadnavirus*, is a dsDNA virus that infects the liver cells of the host, causing acute as well as chronic hepatitis B disease [88]. According to the World Health Organization, in 2015, 887,000 people died due to HBV complications, including cirrhosis and hepatocellular carcinoma, and as many as 227 million people are infected with this virus [89]. Particles from the HBV core are inoculated into the nuclei of liver cells, and then the genomes of this virus are arranged into covalently closed circular DNA (cccDNA). This cccDNA functions as a template for transcription of messenger RNA (mRNA) as well as a 3.5-kb pregenomic RNA (pgRNA). These pgRNAs are used as the template for reverse transcription, resulting in viral genome replication. In other words, the formation of pgRNA during transcription is critical for replication of the HBV genome [90,91,92].

AgNPs with average diameters of 10 nm (Ag10Ns) and 50 nm (Ag50Ns) were assembled in 4-(2-hydroxyethyl)-1-piperazineethanesulfonic acid (HEPES buffer) from AgNO_3_, and these AgNPs attached to the HepAD38 cell line to produce HBV pgRNA, as confirmed by TEM, to inhibit HBV genome replication [93].

### 6.3. Herpes Simplex Virus

As a common agent of infection, herpes simplex virus type 1 (HSV-1) infects humans worldwide with no age limit [94]. HSV-1 is responsible for orolabial ulcers along with a range of other clinical manifestations from asymptomatic cases to severe encephalitis. In 2012, 3.7 million people between 0 and 49 years of age were estimated to be infected, with most infections in Africa, Southeast Asia, and the Western Pacific region. One hundred forty million people, mostly in the Americas, Europe, and the Western Pacific, were estimated to have genital infections based on the assumption of 50% incident infections among 15- to 49-year-olds [95]. HSV-1 is a member of the *Herpesviridae* virus family, also known as human herpes virus 1, and has a 152-kb dsDNA genome encapsulated within an icosahedral capsid with a lipid bilayer envelope [96]. Cell access by HSV-1 depends on interactions between glycoproteins on the HSV-1 envelope and heparin sulfate (HS) moieties on the surfaces of cells [97,98]. Silver nanoparticles enveloped with mercaptoethane sulfonate can mimic HS. These nanoparticles were shown to interact proficiently with HSV-1 to inhibit viral contact with host cells [30].

### 6.4. Human Immunodeficiency Virus-1 (HIV-1)

Sexually transmitted infections are considered a major public health problem worldwide. HIV-1 can be transmitted through blood, semen, pre-ejaculate, rectal fluids, and vaginal fluids. This virus can lead to acquired immunodeficiency syndrome or AIDS. Even after treatment, the human body cannot eliminate HIV, which attacks the human immune system, specifically the CD4 cells (T cells) that help the immune system fight infections [99,100].

Several studies have investigated whether AgNPs can function as antiviral agents against HIV. Condoms can prevent 85% of HIV infections. Nonoxynol-9-coated condoms, however, can induce ulceration and inflammation of the genital mucosa, which might increase the rate of infection. AgNP-coated polyurethane condoms (PUCs) were developed and showed antiviral activity against HIV and HSV infections. Nanoparticles were stable on the PUCs and were not washed away by water. Furthermore, AgNP-coated PUCs had no significant toxic effects on a variety of human cells (HeLa cells, 293T cells, and C8166 T-cells). Thus, condoms coated with AgNPs can prevent HIV-1 infection [101].

In a luciferase-based assay, AgNPs coated with poly N-vinyl-2-pyrrolidone (PVP) interacted with and attached to the gp120 receptor of the HIV-1 envelope. The particles attached to a disulfide bond in the CD4 binding domain of gp120. HIV-1 uses gp120 to interact with the host cell receptors of CD4 cells. The inhibitory effect of AgNPs against the gp120-CD4 interaction was confirmed by enzyme-linked immunosorbent assay (ELISA), which also revealed that AgNPs had antiviral activity in cells infected by HIV-1 [102,103]. High-angle annular dark-field scanning transmission electron microscopy revealed that three types of AgNPs interacted with one another and attached to the gp120 of HIV-1. Foamy AgNPs were found to have a greater antiviral effect than PVP-coated AgNPs and bovine serum albumin (BSA)-conjugated AgNPs [104]. Another study demonstrated the dose-dependent cytoprotective activity of AgNPs. The AgNPs, which were synthesized using HEPES buffer and coated with PVP, protected against HIV-1 infection and also demonstrated anti-HIV-1 activity post-infection [105].

### 6.5. Influenza A

Influenza A viruses are divided into subtypes on the basis of hemagglutinin (HA) and neuraminidase (NA). There are 18 HA subtypes and 11 NA subtypes. Many combinations of HA and NA proteins are possible. The “H7N2 virus” is the influenza A virus that normally infects birds. However, in humans, infection by this virus is accompanied by fever, chills, nonproductive cough, sore throat, runny or stuffy nose, muscle and body aches, headache, and fatigue [106]. HA inhibition tests and embryonated inoculation assays have revealed that AgNPs can inhibit the HA of chicken red blood cells infected with H7N2. TEM and flow cytometry have shown that AgNPs diminish the virus-induced apoptosis of Madin-Darby canine kidney (MDCK) cells. The AgNPs used in these experiments were 5 to 20 nm in size with an average diameter of 10 nm, and their cytotoxicity toward MDCK cells was measured by the 3-(4, 5-dimethylthiazol-2-yl)-2, 5-diphenyltetrazolium bromide (MTT) assay [107]. Influenza virus-infected MDCK cell cultures were used in another experiment to investigate the inhibitory potential of AgNPs. The study revealed that AgNPs with no coating were able to destroy the viral membrane glycoproteins that the virus uses to infect host cells. This phenomenon was assessed by the HA assay, and the size of the AgNPs was 5 to 20 nm [108]. Another study lso found effective results in the inhibition of various Influenza A viruses by Ag-SiO_2_ particles, where the size of the AgNPs was 30 nm [109]. Moreover, a recent study reported inhibition of Influenza H5N1 using a combination of Zn_2_ and AgNPs, illustrating the potential of AgNPs for therapeutic applications [110].

### 6.6. Noroviruses

Noroviruses, members of the family *Caliciviridae* and genus *Norovirus*, are non-enveloped positive-sense, single-stranded RNA ((+)ssRNA) viruses that contain approximately 7.5 kb of genetic material surrounded by capsid proteins [111]. These viruses are referred to as “winter vomiting bugs” in the UK and Ireland. Noroviruses are the most common cause of viral gastroenteritis [112]. Nausea, vomiting, abdominal pain, low-grade fever, malaise, and muscle pain are common symptoms of norovirus infection. Human norovirus (NoV) causes about 267 million people to fall sick a year, and more than 200,000 deaths worldwide each year are caused by these viruses [113].

A recent study investigated the antiviral activity of different doses (25, 50, and 100 μg/m) of AgNPs of various sizes (10, 75, and 110 nm in diameter) against feline calicivirus (FCV) [114]. Cultivating human NoV in cell culture is difficult. Hence, FCV, which has a similar genomic organization and structure to human NoV, is used as a surrogate [115]. Only AgNPs with a diameter of 10 nm effectively decrease FCV titer. The size of FCV (27–40 nm) is comparable to that of 10-nm AgNPs, and the interaction that occurs might be due to the similar sizes of the nanoparticles and virus. Another study reported a reduction in FCV VP1 viral capsid protein level in response to treatment with 10-nm AgNPs. The FCV VP1 protein is essential for attachment to functional receptor molecules in permissible cells [116]. Thus, 10-nm-diameter AgNPs can reduce norovirus activity.

### 6.7. Poliovirus

Poliomyelitis, usually called polio, is caused by a human enterovirus known as poliovirus. The virion of poliovirus, a member of the *Picornaviridae* family and *Enterovirus* genus, is a (+)ssRNA virus with a genome ~7500 nucleotides in size and containing three capsid proteins (VP1, VP2, and VP3) [117]. It can interact with the host in two ways. If the central nervous system is infected, paralysis can result. If the central nervous system is not infected, only minor illness results. This virus is transmitted mainly by the fecal-oral route [118].

A recent study showed that electrochemically synthesized AgNPs had activity against poliovirus-infected human rhabdomyosarcoma (RD) cells. The AgNPs had no cytopathic effects (CPEs) on RD cells at levels up to 100 ppm. Their antiviral activity was assessed at 3.13 ppm after 30 min at the viral concentration of 1 tissue culture infectious dose 50 (TCID_50_) and after 60 min at 10 TCID_50_. During this time, cell viability was as high as 98%, and no CPE was observed. However, significant CPEs were observed in RD cells after 48 h of infection at a 100 TCID_50_ concentration. The particles used in the mixture of AgNPs and poliovirus were quasi-spherical in shape with a mean size of about 7.1 nm. These studies have contributed to our understanding of the cytotoxicity of pure AgNPs to RD cells [119,120].

### 6.8. Respiratory Syncytial Virus

Respiratory syncytial virus (RSV), an envelope virus, is a member of the *Paramyxoviridae* family and *Orthopneumovirus* genus with a 15.2-kb non-segmented genome comprised of (−)ssRNA [121]. RSV is the major cause of lower respiratory illnesses, including pneumonia and bronchiolitis, among neonates and infants. Hence, the number of clinical trials of vaccines against RSVP is growing, and there is hope that an RSV vaccine will be available on the commercial market within the next 5–10 years [122]. In addition to vaccinations, AgNPs coated with PVP can block RSV infection by 44%. However, AgNPs conjugated with recombinant RSV fusion (RF) and BSA did not yield significant results based on TEM observations. The authors of that study hypothesized that the small size and uniformity of the PVP-coated nanoparticles (4–8 nm) allowed effective binding to the G protein of the RSV virus compared to the BSA- and RF-conjugated nanoparticles (3–38 nm), and that this binding capability was critical for viral inhibition. Trypan blue exclusion studies showed that all three types of AgNPs had less than 20% cytotoxicity up to a concentration of 100 μg/mL after the nanoparticles were combined with RSV and then mixed with human laryngeal epithelial type 2 (HEp-2) cells [49]. In another study, curcumin-based cAgNPs were shown to inhibit RSV. TCID_50_ assays revealed that cAgNPs added to HEp-2 cells reduced the viral titers of RSV at different concentrations, with no toxicity to the host cells [123].

### 6.9. Rift Valley Fever Virus

Rift Valley fever virus (RVFV), members of the family *Paramyxoviridae* and genus *Phlebovirus*, are spherical enveloped (−)ssRNA viruses that contain approximately 11.5 kb of genetic material surrounded by capsid proteins. RVFV is a mosquito-borne pathogen that causes serious disease in ruminants and is transmitted frequently to humans in subsequent epizootic outbreaks. RVFV causes an influenza-like illness in people, but it can also cause more serious consequences with high morbidity and death. For decades, the disease was confined to Sub-Saharan Africa, but in recent years, there has been a dramatic increase in the number of outbreaks, with cases reported in the Arabian Peninsula, Egypt, and some Indian Ocean islands, confirming the disease’s potential to spread worldwide [124,125]. Because there is no existing treatment or licensed Rift Valley fever vaccine(s) for human use, innovative techniques capable of inhibiting viral multiplication and transmission are required for effective disease control. Borrego et al. [126] tested the antiviral activity of AgNPs (as Argovit™) against RVFV. Although the efficacy of silver nanoparticles to suppress an ongoing RVFV infection was restricted, incubating the virus with Argovit before infection resulted in promising results to reduce infection in vitro and in vivo, suggesting that silver nanoparticles could be used to reduce RVFV infection.

### 6.10. SARS-CoV-2

Since the early 20th century, the *Coronaviridae* virus family has impacted humankind, and rapid mutations of this virus in a short period of time have hindered the development of a special therapy to limit its spread and death count [127,128,129]. SARS-CoV-2 was discovered originally in December 2019, in Wuhan City, Hubei Province, China, causing a global epidemic that affected all six continents and infecting 186,284,781 people of all ages as of July 9, 2021. SARS-CoV-2, an envelope virus, is a member of the *Coronaviridae* family and *Betacoronavirus* genus with a ~32-kb genome comprised of (+)ssRNA [130,131]. SARS-CoV-2 is linked physiologically to the body by means of epithelial cells that contain sialic acids on the surface and bind to galactose α-2,6. This virus often enters the body through the epithelial cells that line the human trachea, which contain mostly carbohydrates and have a α-2,6 bond [132,133,134].

In [29], researchers investigated an abundance of AgNPs of various sizes and concentrations and showed that inhibition of extracellular SARS-CoV-2 particles with 10-nm diameters was effective between 1 and 10 ppm, while the cytotoxic effect was observed at levels above 20 ppm. In another study [135], a cytotoxicity assay was conducted using polyacrylic acid, but the MTT assay was safest as a higher concentration of cells was necessary to kill 50 percent of the viable cells. AgNPs also have significant antiviral activity against MERS-CoV, with a viral suppression of 48.3 percent at 0.0625 L.

### 6.11. Chikungunya Virus

Chikungunya is a mosquito-transmitted viral disease. Chikungunya virus (CHIKV) is a member of the family *Togaviridae* and genus *Alphavirus* with an ~11.8-kb genome comprised of enveloped single-strand RNA virus. CHIKV usually is a quiet virus; however, a recent mutation in the E1 gene at position A226V has broadened its vector range. The CHIKV resurfaced between 2000 and 2008, when an east African strain spread over the Republic of Congo and other neighboring islands [136]. Many recent outbreaks have been linked to the mutant form of CHIKV, such as the 1.3 million cases reported in India in 2006 [137]. The WHO reported the first local CHIKV transmission in the Americas in 2013–2014 [138]. According to epidemiologic and disease dynamics research, the virus affects roughly 3 million individuals each year, and 1.3 to 2.7 billion people live in places where CHIKV transmission is a danger [138,139]. As there is no specific cure for Chikungunya and the disease has such a large impact, scientists recommend using monoclonal antibodies, designer molecules, and nucleic acid modifiers [36].

Sharma et al. [140] examined the biological synthesis of AgNPs from *Andrographis paniculata*, *Phyllanthus niruri*, and *Tinospora cordifolia*, as well as their antiviral effects against the Chikungunya virus. Among them, *A. paniculata* AgNPs were the most effective in an in vitro antiviral experiment based on degree of inhibition of CPE, followed by *T. cordifolia* and *P. niruri* AgNPs. The antiviral assay results were validated by a cell viability test using MTT dye, which demonstrated that *A. paniculata* AgNPs completely suppressed the virus. When CHIKV-infected cells were treated with *A. paniculata* AgNPs at MNTD and 1/2 MNTD, cell viability increased considerably from 25.69% to 80.76 and 66.8%, respectively. The same research group also reported that *Psidium guajava* AgNPs were effective to control CHIKV [141]. These findings suggest that using plant-based AgNPs as antiviral medicines is viable and could provide alternative therapeutic options for viral infections for which no particular antivirals or vaccines are currently available.

### 6.12. Bunyamwera Orthobunyavirus

*Bunyavirales* is the most diversified order of negative-sense RNA viruses and includes viruses from plants, animals, and humans [142]. These viruses are named after the Bunyamwera virus (BUNV), the type species of the *Orthobunyavirus* genus discovered in Uganda in 1943 (a neurotropic isolated virus). In many mammals, including humans, the illness associated with BUNV infection causes moderate symptoms such as fever, joint pain, and rash; however, in immunocompromised individuals, the infection can cause severe encephalitis [143]. The majority of bunyaviruses is arthropod-borne viruses that, along with other arboviruses like dengue, Zika, and Chikungunya, are spreading rapidly to new geographical areas. This is due to rapid spread of arthropod vectors, which is aided by global warming and movement of human populations, which enter new wild environments, coming into contact with vectors [144,145]. Despite their expanding worldwide significance, there are no licensed treatments or vaccines to combat bunyaviral infections [146]. García-Serradilla and Risco [147] investigated the antiviral capacity of AgNPs in BUNV-infected Vero cells and discovered that AgNPs are effective inhibitors of BUNV infection in these cells, causing changes in the ultrastructure of BUNV spherules/ROs and significantly lowering virus titers in cell supernatants.

### 6.13. White Spot Syndrome Virus

White spot syndrome virus (WSSV), members of the family *Nimaviridae* and genus *Whispovirus*, are pleomorphic, enveloped, negative-sense ssRNA viruses that contain approximately 300 kb of genetic material surrounded by capsid proteins [148,149,150]. WSSV is the primary agent of shrimp white spot disease, which has impacted the shrimp business [151,152]. The WSSV was discovered in Mexico in 1999, but it was not identified as a disease until 2004, in shrimp farms on Mexico’s northwest beaches [153]. Despite the fact that antivirals have been introduced to the market, and biopharmaceuticals were also reported as immunostimulant factors, no antiviral or immunostimulant agent capable of decimating viral infections against shrimp disease has been developed [154,155].

Ochoa-Meza et al. [156] reported that 12 ng/mL silver nanoparticles resulted in 20% survival of treated diseased shrimp, whereas the same quantity provided to healthy shrimp resulted in no histological evidence of damage. The lipopolysaccharide- and β-1,3-glucan-binding protein is a critical gene in the shrimp immune response, and its activation is caused most likely by identification of AgNPs coated with certain pathogen-associated molecular pattern recognition proteins in shrimp. These findings demonstrated that a single treatment with a small amount of AgNPs was capable of enhancing the response of the shrimp immune system without causing harmful effects in healthy shrimp.

### 6.14. Zika Virus

Zika virus (ZIKV), members of the family *Flaviviridae* and genus *Flavivirus*, are spherical, enveloped, positive-sense, single-strand RNA viruses that contain approximately 10 kb of genetic material surrounded by capsid proteins [157,158]. Mosquito management is crucial in preventing mosquito-borne disease outbreaks, since this disease is spread by daytime-active *Aedes* mosquitoes [159]. Synthetic pesticides like organophosphates and pyrethroids, as well as insect growth regulators like diflubenzuron and methoprene, are powerful components for mosquito control [160]. However, these treatments have resulted in a slew of health and environmental concerns, including increased resistance and disruption of the ecosystem’s natural control systems [161]. With the recent introduction of AgNPs, mosquito vector control might become more effective and environmentally safe. In [35], *Aquilaria sinensis* essential oil and Pogostemonis Herba essential oil of *Pogostemon cablin* were used to bio-fabricate AgNPs in a single step and at a low cost. Even at low dosages, the produced AgNPs demonstrated considerable larvicidal and pupicidal toxicity against the *Aedes albopictus* mosquito. Furthermore, histological tests revealed that AgNPs had the ability to harm mosquito larvae digestive systems and midgut cells. Available data regarding these viruses and the effect of AgNPs against these viruses are represented in Table 1 and Table 2.

## 7. Toxicity and Safety Issue of AgNPs

Nanomaterials have been reported to be very cytotoxic to mammalian cells because of their interaction with biomolecules that produce reactive oxygen through defense mechanisms that cause damage to lipids, proteins, and DNA by oxidation [190]. The toxicity of AgNPs is considered to be linked with their direct binding to the viral protein surface [93]. As a result, suitable surface modifications can be accomplished by determining the precise interaction location. The primary parameters determining the toxic effects of AgNps in organisms are characterized according to route of exposure, namely entry, concentration, duration and inherent toxicity of the AgNps, bioavailability, and body accumulation [191].

Three basic modes of AgNP exposure are inhalation, cutaneous exposure, or oral exposure. The largest AgNPs can be expelled when entering the body, whereas tiny AgNPs can be deposited in the lung and reach other organs via the bloodstream. According to histopathological investigation, no significant changes were observed after applying AgNPs in the nasal cavities [192], liver [193], or lungs [194], among other organs, with sizes of 15–30 nm and concentrations of 0.5–381 g/m^3^ [195]. However, at high quantities above 2.9 mg/m^3^, AgNPs can cause brain damage [196].

The cytotoxicity of AgNPs increases with size from 20–100 nm and dosage from 0.1–1000 mg/kg in parenteral injections, causing lung [197], renal [198], liver [199], and brain lesions [200]. The toxicity of AgNPs from oral consumption is intermediate. NPs with sizes ranging from 3–60 nm were tested previously [201], although the size of the NPs was not as important as the dose for oral toxicity. Dosages ranged from 0.5 μg to 500 mg/L. Dosages of 10 mg/kg resulted in weight loss [202], doses greater than 300 mg/kg resulted in liver problems [203], and doses greater than 1000 mg/kg resulted in oxidative stress [203,204]. Nonetheless, determining the toxicity of AgNPs in humans is challenging due to a lack of exposure research [191]. However, investigations of NP treatment scenarios should be expanded, particularly those concentrating on levels in the lungs, because inhalation is the most common mode of exposure. In addition, different in vivo studies also revealed that AgNPs those are administered by ingestion or inhalation can damage different organs like brain, kidneys and liver (Table 3).

To lessen toxicity, the metal surfaces of the nanoparticles must be modified such that they do not connect directly to cells. In addition, their concentration in the interior of a cell compartment should not be high. Specified by the National Institute for Occupational Safety and Health (NIOSH) [205], nanocomposites and nanomaterials with a surface coating or containing nanostructures rarely pose a threat to the associated workers due to possessing a non-inhalable material size. Having said that, sometimes during the manufacturing process there generates inhalable sizes of nanomaterials as well making them unsafe for the handlers [205]. The human-engineered nanomaterials are far more alarming than the natural and incidental [206] ones. The processes for evaluating threats from the man-made nanoparticles consists of recognizing and specifying those materials, investigating exposure level, and identifying related risks. The important processes in assessing the danger of manmade nanomaterials are similar to those in assessing the risk of conventional substances which are hazard identification and characterization, assessment of exposure, and risk characterization. While human-fabricated nanoparticles have opened a new door for working in nanoscale, they have also exacerbated the situation regarding their safe usage and risk factors due to their unconventional characteristics such as size and diversity. These characteristics vary because of their generation methods and used substances [207]. However, several government, non-government and research approaches are being made on how to identify, specify, and tackle these risk issues in order to keep the related people safe. Although nanoparticle research is progressing, the specific mechanisms of nanomaterial effects are unknown, necessitating improvements in terms of safety to maximize therapeutic breakthroughs.

## 8. Antiviral Application of AgNPs

The usage of AgNPs is in the preclinical stage, limiting their application. Overall, AgNPs fight against a virus in the human body in two primary ways: vaccination and oral intake. Vaccination is one of the most efficient ways to prevent infectious diseases while also limiting healthcare expenses [220]. Vaccines are bioparticles that enhance the delivery of acquired immunity against infectious illnesses. Virus-like particles, attenuated viruses, or protein-subunit antigens are used commonly in vaccines to promote an immune response against infectious illnesses. The immunoactivity of natural and artificial nanoparticles has been studied since the emergence of nanotechnology [221]. Several researchers have investigated the impact of AgNPs on inflammatory action, in which silver reacts and stimulates immune cells under different circumstances [222]. In 2013, Xu et al. [223] investigated the immunological activity of AgNPs in vitro and in vivo using bovine serum albumin and ovalbumin as antigens. AgNPs boosted the generation of serum antigen-specific IgG and antigen-specific IgE after subcutaneous immunization of mice. According to another study, increased AgNPs also caused an increase in inflammatory cytokine release in rat alveolar macrophages [224]. When taken orally, the minimal inhibitory concentration (MIC) must be determined for colloidal AgNPs. A recent study indicated that the MIC is highly sensitive to smaller nanoparticles, which contain a higher number of particles at a certain weight, resulting in a higher density of particles that can best interact with the pathogen at a low MIC value [225].

## 9. Conclusions and Future Prospects

AgNPs have been investigated extensively over decades due to their unique physical, chemical, optical, and electrical properties, all of which are related to AgNP features, particularly size and shape. Most of the studies found that an AgNP size less than 20 nm was necessary for an effective result against viruses. Current research suggests that the most studied and successfully inhibited virus using AgNPs is Influenza, A. Based on recent developments, utilization of nanoparticles can be regarded as a viable option for antiviral applications. Although the mechanisms of action of AgNPs are not entirely understood, studies suggest their effectiveness. Their antiviral action against some viruses has been examined widely and proven to be successful enough to broaden this study area to include nano-treatments effective against a wide spectrum of viruses.

Climate change, poverty, malnutrition, and other factors contribute to infections caused by microbes, especially viruses. As obligate intracellular parasites, viruses often interact with host cells through different types of receptor-ligand interactions. To develop antiviral drugs, viral diseases are being researched intensively, including their life cycle complexities, variations in replication in various organelles or subcellular compartments and related dynamics, latent infection potentiality in inaccessible bio-compartments, and evolution of drug resistance, among other aspects. The development of new antiviral agents is difficult and time-consuming; however, recent advancements in nanomedicine have revealed potential therapeutic effects of AgNPs as antiviral agents. Several hurdles related to biocompatibility, toxicity, and potential side effects need to be overcome before AgNPs can be used as therapeutic agents in humans. Further scientific studies are needed on the development of AgNPs for use as antiviral agents.

## Figures and Tables

**Table 1:** Properties of viruses included in AgNP research.

Virus	Family/Genetic Material	Capsid/Coat	Genome Size	Virion Diameter	References
Adenovirus type 3	Adenoviridae/dsDNA	Icosahedral/Non-enveloped	~30–40 kb	~70 to 100 nm	[162,163]
HBV	Hepadnaviridae/dsDNA-RT	Icosahedral/Enveloped	~3.2 kb	22–4 nm	[164,165]
HSV-1 (DNA)	Herpesviridae/dsDNA	Icosahedral/Enveloped	~152 kb	155 to 240 nm	[96,166,167]
HSV-1 (RNA)	Retroviridae/ssRNA-RT	Conical Complex/Enveloped	~9.2 kb	∼145 nm/Positive-sense	[168,169]
Influenza A	Orthomyxoviridae/ssRNA	Helical Complex/Enveloped	~7.12–18.73 kb	10–15 nm/Negative-sense	[170,171]
Norovirus	Calicivirida/ssRNA	Icosahedral/Naked	~7.5–7.7 kb	~38 nm/Positive-sense	[111,172,173]
Poliovirus	Picornaviridae/ssRNA	Icosahedra/Naked	~7.5 kb	31 nm/Positive-sense	[117,174]
RSV	Paramyxoviridae/ssRNA	Helical/Enveloped	~15.2 kb	100–1000 nm/Negative-sense	[175,176]
RVF	Phenuiviridae/ssRNA	Spherical/Enveloped	~11.5 kb	80–120 nm/Negative-sense	[177]
SARS-CoV-2	Coronaviridae/ssRNA	Coiled Helix/Enveloped	~32 kb	50–200 nm/Positive-sense	[130,131]
Chikungunya virus	Togaviridae/ssRNA	Icosahedral/Enveloped	~11.8 kb	70 nm/Positive-sense	[178,179,180,181]
BUNV	Peribunyaviridae/ssRNA	Pleomorphic/Enveloped	~6.9 kb	108 ± 8 nm/Negative-sense	[143,182]
WSSV	Nimaviridae/dsDNA	Ovoid/Enveloped	~300 kb	70–167 nm	[148,149,150]
ZIKV	Flaviviridae/ssRNA	Spherical/Enveloped	~10 kb	50 nm/Positive-sense	[157,158]

**Table 2:** AgNPs and their inhibitory actions against viral entities.

Virus	Synthesis	Characterization	Coating/Size	Target	Inhibitory Actions	References
Adenovirus type 3	Chemical	XRD, TEM	Uncoated/~11.4 ± 6.2 nm	Viral concentration of TCID_50,_ HeLa cells	Directly damaged Ad3 particles	[87]
HBV	Chemical	SPR, XRD, TEM, UV-Vis	Uncoated/~10 nm, ~50 nm	HepAD38 cells	Bound to HBV dsDNA and reduced extracellular DNA formation and intracellular RNA formation	[93]
HSV-1 (DNA)	Sonochemical	TEM, XPS	Coated, MES/4 nm, 13 nm, 33 nm, and 46 nm	Vero cells, GMK-AH1 cells, mouse keratinocyte 291.03C cells, α-MEM cells	Infection was mostly blocked or reduced	[30,183]
HSV-1 (RNA)	Chemical	EM, DRS, FTIR, EDXS	Uncoated/30–60 nm	C8166 T, HeLa β-gal-CD4 + -CCR + cells	Decreased infectivity as observed by counting the number of GFP+ cells or syncytium formation	[79]
Influenza A	Chemical	XRD, TEM	Uncoated/1–400 nm	Hemagglutinin, MDCK cells, Vero, MDFK, MDCK Mice BALB/c	Reduced or completely inhibited agglutinated erythrocytes and inhibited apoptosis in MDCK cells	[107,108,109,184,185,186,187,188]
Norovirus	Chemical	TEM, DLS	Uncoated/10, 75, 110 nm	FCV	Inactivation of FCV might be due to physical interactions with VP1	[114]
Poliovirus	Electrochemical	UV-Vis, EDXS, TEM	Uncoated/4 to 9 nm	Viral concentration of TCID_50_	Prevented viral particle binding to the receptors of RD cells	[189]
RSV	Chemical	UV-Vis, DLS, SEM, TEM	Curcumin, and uncoated/10, 19.72 ± 0.54 nm	Viral concentration of TCID_50,_ HEp-2 cells, A549 (type II) and HEp-2 Mice BALB/c	Inactivated RSV directly before entering cells	[82,83,123]
RVF	Chemical	--	Coated/35 nm	Vero cell cultures and in type-I interferonreceptor deficient mice (IFNAR −/− mice)	Before the infection, reduction of infectivity	[126]
SARS-CoV-2	Photochemical	UV-VIS, TEM, SEM, zeta potential analysis	Coated/10–30 nm, 2–15 nm	Vero E6 cells (10^5^ cells/mL), Calu-3 cell lines	Extracellular viruses are inhibited by silver nanoparticles because they prevent viral entrance	[29]
Chikungunya virus	Biological(plant)	UV-Vis, FTIR, SEM, DLS, zeta potential measurements	Coated/70–120 nm	Vero cells	Inhibition occurs as AgNps reduce/stop the replication of the Chikungunya virus in cell-line and in silico studies	[140,141]
BUNV	--	TEM, correlative light and electron microscopy	Coated/10 nm	Vero cells (CCL-81)	Potent inhibitors caused changes in the ultrastructure virus and significantly lowered virus titers in cell supernatants.	[81]
WSSV	Chemical	TEM	Coated/35 nm (avg)	*Penaeus vannamei* shrimp	LGBP levels rise as a result of the recognition of AgNPs or their contact with the WSSV viral envelope, which activates PAMP recognition proteins.	[156]
ZIKV	Biological(plant)	UV–vis, SEM, TEM, EDS, XRD, FTIR	Coated/15–55 nm, 16–87 nm	*Aedes albopictus* (larvae and pupae)	The larvae were severely affected, with substantial damage to the midgut epithelial cells	[35]

**Table 3:** Toxicity of AgNPs in in vivo models.

Route of Administration	Model	Size of the Particle	Dose	Effect	Reference
Oral	Male Wistar rats	10 ± 4 nm (CT-capped)	0.2 mg/kg	Induced oxidative stress in brain but not in liver	[208]
Inhalation	Sprague–Dawley rats	18 nm	0.6 × 106 particle/cm3, 49 μg/m3(low dose), 1.4 × 106 particle/cm3, 133 μg/m3 (middle dose) and 3.0 × 106 particle/cm3, 515 μg/m3 (high dose)	Silver accumulated in lung, liver, Brain, Kidneys with increase of bile duct hyperplasia in AgNP-exposed liver	[209]
Oral	F344 rats	56 nm	30, 125, 500 mg/kg	Accumulation of silver in kidneys was gender-dependent, with a 2-fold increase in female kidneys.	[210]
Intratracheal instillation	Female Wistar rats	50 nm; 200 nm (PVP-coated)	0.1875, 0.375, 0.75, 1.5, 3 mg/kg	Accumulation of Ag in liver, spleen and kidney with inflammation in lung.	[211]
Oral	Male Sprague Dawley rats	20 nm	820 mg/kg	AgNPs induces liver and cardiac oxidative stress	[212]
Inhalation	Male C57Bl/6 mice	10 nm (PVP-coated)	3.3 ± 0.5 mg/m3 or 31 µg/g lung	Minimal pulmonary toxicity.	[213]
Oral	Sprague Dawley rats	10 nm; 75 nm; 110 nm	9, 18, 36 mg/kg	No toxic effect on blood, reproductive and genetic system tested was observed.	[214]
Intratracheal instillation	BALB/C mice	10 nm	0.05, 0.5, 5 mg/kg	Oxidative stress, DNA damage, apoptosis in heart	[215]
Oral	Male Sprague Dawley rats	20–30 nm (PVP-coated)	50, 100, 200 mg/kg	High dose of AgNPs induced hepatocellular damage by increased ROS production	[216]
Inhalation	BrownNorway and Sprague–Dawley rats	15 nm	8, 28 µg	Accumulated in lungs with production of proinflammatory and pro-neutrophilic cytokines.	[217]
Intratracheal instillation	Male Sprague–Dawley rats	20 nm (CT-capped)	1 mg/kg	Cardiac ischemic-reperfusion injury.	[218]
Inhalation	Female C57BL/6 mice	18–20 nm	3.80 × 107 part. /cm^−3^	Increased number of resorbed fetuses associated with reduced estrogen plasma levels	[219]

## Data Availability

Not applicable.
